# Therapeutics of Wounds in the Joints and Ligaments

**Published:** 1898-05

**Authors:** 


Therapeutics of Wounds in the Joints and Ligaments.—At a general meeting of the Brunswick veterinarians Dr. Schrader, of Helmstadt, reported in regard to his therapeutics of wounds in the joints and tendons. Formerly he had always tried to heal the wound by firing, tannin, ferrum sesquichlor, with collodion, etc. However, he never succeeded with these means, and for this reason he decided to treat wounds in the joints and tendons by making an incision, irrigating it with antiseptics, and powdering it with iodoform tannin. He has reached very satisfactory results by this means. Schrader described the course of healing in several cases where the tendons of the pedal bone, the elbow, and the fetlocks had been injured. Dr. Bertram said that he could heartily recommend, as very effective in such cases, a powder consisting of iodoform, tannin, and camphor. Saake said that he had found that a sublimate, 1 to 5000, with which the wounds could be washed out, brought better results than the old method of treatment.—*Thierärzt. Centralblatt*, February 20, 1898.

